# Supplementary material for: Discerning Transcriptomic and Biochemical Responses of Arabidopsis thaliana Treated with the Biofertilizer Strain Priestia megaterium YC4-R4: Boosting Plant Central and Secondary Metabolism
Source: Plants (Basel). 2022 Nov 10;11(22):3039. doi: 10.3390/plants11223039 (PMC9697256; doi:10.3390/plants11223039)
Supplement: Supplementary file 1 [file plants-11-03039-s001.zip › Ref. 15.pdf]

frontiers in Agronomy

ORIGINAL RESEARCH
published: 17 June 2021
doi: 10.3389/fagro.2021.665798

Amelioration of Saline Stress on Chia (*Salvia hispanica* L.) Seedlings Inoculated With Halotolerant Plant Growth-Promoting Bacteria Isolated From Hypersaline Environments

María Florencia Yañez-Yazlle[1,2†], *Neli Romano-Armada*[1,3†], *Verónica Beatriz Rajal*[1,3,4†] and *Verónica Patricia Irazusta*[1,2*†]

[1] Instituto de Investigaciones para la Industria Química (INIQUI), Universidad Nacional de Salta (UNSa), Consejo Nacional de Investigaciones Científicas y Técnicas (CONICET), Salta, Argentina, [2] Facultad de Ciencias Naturales, Universidad Nacional de Salta (UNSa), Salta, Argentina, [3] Facultad de Ingeniería, Universidad Nacional de Salta (UNSa), Salta, Argentina, [4] Singapore Centre for Environmental Life Sciences Engineering (SCELSE), School of Biological Sciences, Nanyang Technological University (NTU), Singapore, Singapore

OPEN ACCESS

Edited by:
Antonio Rafael Sánchez-Rodríguez,
University of Cordoba, Spain

Reviewed by:
Sheng Qin,
Jiangsu Normal University, China
Ana María García-López,
University of Seville, Spain

***Correspondence:**
Verónica Patricia Irazusta
irazustaveronica@gmail.com

†ORCID:
María Florencia Yañez-Yazlle
orcid.org/0000-0002-1623-5715
Neli Romano-Armada
orcid.org/0000-0003-3148-2041
Verónica Beatriz Rajal
orcid.org/0000-0002-2290-8920
Verónica Patricia Irazusta
orcid.org/0000-0001-7436-8543

Specialty section:
This article was submitted to Plant-Soil Interactions, a section of the journal Frontiers in Agronomy

Received: 08 February 2021
Accepted: 20 May 2021
Published: 17 June 2021

Citation:
Yañez-Yazlle MF, Romano-Armada N, Rajal VB and Irazusta VP (2021) Amelioration of Saline Stress on Chia (Salvia hispanica L.) Seedlings Inoculated With Halotolerant Plant Growth-Promoting Bacteria Isolated From Hypersaline Environments. Front. Agron. 3:665798. doi: 10.3389/fagro.2021.665798

The rhizosphere and microbiome of halotolerant plants could be crucial for alleviating salinity stress during plant growth. The aims of this work were (1) to isolate bacteria from rhizosphere and bulk soil samples from the Salar del Hombre Muerto (Catamarca, Argentina), (2) to characterize different plant growth-promoting (PGP) activities produced by these bacterial isolates, and (3) to evaluate their effect on the initial growth of chia (*Salvia hispanica* L.) under saline stress. A total of 667 microorganisms were isolated, using different culture media with NaCl, and their abilities for nitrogen fixation, phosphate solubilization, siderophores production, and indole-3-acetic acid production were evaluated. Thirteen strains were selected for showing all the tested PGP activities; they belonged to the genera *Kushneria*, *Halomonass*, *Pseudomonas*, *Planomicrobium*, and *Pseudarthrobacter*. The strains *Kushneria* sp. and *Halomonas* sp. showed the highest salinity tolerance (from 50 to 2,000 mM NaCl) and biomass and biofilm production. Chia seeds were treated with six of the first 13 selected strains to evaluate their plant growth-promoting effect under saline stress (without and with 50 and 100 mM NaCl). *Halomonas* sp. 3R.12 and *Kushneria* sp. T3.7 produced heavier seedlings with a balanced shoot/root length ratio, while *Pseudomonas* sp. AN23 showed the best effect upon chia seedlings, with a morphological response similar to non-stressed seedlings. On the other hand, seedlings displayed no responses when inoculated with *Planomicrobium* sp. 3S.31 and *Pseudarthrobacter* sp. ER25. This study contributes to the knowledge on microorganisms from hypersaline environments as plant growth promoters for their use in the production of salt-sensitive crops, among other potential uses.

Keywords: halotolerant bacteria, nitrogen fixation, phosphate solubilization, siderophores production, indole-3-acetic acid production, biofilms production, *Kushneria, Halomonas*

Mukhtar, S., Mehnaz, S., and Kauser, A. (2019). Microbial diversity in the rhizosphere of plants growing under extreme environments and its impact on crop improvement. *Environ. Sustain.* 2, 329–338. doi: 10.1007/s42398-019-00061-5

Muñoz, L. A., Cobos, A., Diaz, O., and Aguilera, J. M. (2013). Chia seed (*Salvia hispanica*): an ancient grain and a new functional food. *Food Rev. Int.* 29, 394–408. doi: 10.1080/87559129.2013.818014

Murashige, T., and Skoog, F. (1962). A revised medium for rapid growth and Bio assays with Tobacco tissue cultures. *Physiol. Plant.* 15, 474–497. doi: 10.1111/j.1399-3054.1962.tb08052.x

Nagar, S., Kumar, A., Shalini, S., Mary, A. S., Pramod, C., and Rai, K. (2018). Aerobic biodegradation of HMX by *Planomicrobium flavidum*. *3 Biotech.* 8:455. doi: 10.1007/s13205-018-1479-5

Navarro-Torre, S., Mateos-Naranjo, E., Caviedes, M. A., Pajuelo, E., and Rodríguez-Llorente, I. D. (2016). Isolation of plant-growth-promoting and metal-resistant cultivable bacteria from Arthrocnemum macrostachyum in the Odiel marshes with potential use in phytoremediation. *Mar. Pollut. Bull.* 110, 133–142. doi: 10.1016/j.marpolbul.2016.06.070

Orona-Tamayo, D., Valverde, M. E., and Paredes-López, O. (2017). "Chia — The new golden seed for the 21st century : nutraceutical properties and technological uses," in *Sustainable Protein Sources*, eds S. R. Nadathur, J. P. D. Wanasundara, and L. Scanlin (Elsevier Inc.), 265–281. doi: 10.1016/B978-0-12-802778-3.00017-2

Palacio-Rodríguez, R., Coria-Arellano, J. L., López-Bucio, J., Sánchez-Salas, J., Muro-Pérez, G., Castañeda-Gaytán, G., et al. (2017). Halophilic rhizobacteria from Distichlis spicata promote growth and improve salt tolerance in heterologous plant hosts. *Symbiosis* 73, 179–189. doi: 10.1007/s13199-017-0481-8

Panda, A., and Parida, A. K. (2019). "Development of salt tolerance in crops employing halotolerant plant growth– promoting rhizobacteria associated with halophytic rhizosphere soils," in *Saline Soil-Based Agriculture by Halotolerant Microorganisms*, eds M. Kumar, H. Etesami, and V. Kumar (Singapore: Springer Nature Singapore Pte), 75–101. doi: 10.1007/978-981-13-8335-9_4

Pérez Brandán, J., Curti, R. N., and Acreche, M. M. (2020). Developmental responses of chia (*Salvia hispanica*) to variations in thermo-photoperiod : impact on subcomponents of grain yield. *Crop. Pasture Sci.* 71, 183–189. doi: 10.1071/CP19218

Pilet, P. E., and Chollet, R. (1970). Sur le dosage colorime'trique de l'acide indolylace'tique. *Comptes Rendus l'Acad. Sci.* 271, 1675–1678.

Pospiech, A., and Neumann, B. (1995). A versatile quick-prep of genomic DNA from gram-positive bacteria. *Trends Genet.* 11, 217–218. doi: 10.1016/S0168-9525(00)89052-6

Raimondi, G., Rouphael, Y., Di Stasio, E., Napolitano, F., Clemente, G., Maiello, R., et al. (2017). Evaluation of *Salvia hispanica* performance under increasing salt stress conditions. *Acta Hortic.* 1170, 703–707. doi: 10.17660/ActaHortic.2017.1170.88

Reginato, M., Sgroy, V., Llanes, A., Cassán, F., and Luna, V. (2012). "The American halophyte prosopis strombulifera, a new potential source to confer salt tolerance to crops," in *Crop Production for Agricultural Improvement*, eds M. Ashraf, M. Öztürk, M. S. Aqeel Ahmad, and A. Aksoy (Dordrecht: Springer Science+Business Media B.V), 20. doi: 10.1007/978-94-007-4116-4_5

Romano Armada, N., Doccula, F. G., Candeo, A., Valentini, G., Costa, A., and Bassi, A. (2019). *In vivo* light sheet fluorescence microscopy of calcium oscillations in *Arabidopsis thaliana*. *Calcium Signalling. Methods Mol. Biol.* 1925, 87–101. doi: 10.1007/978-1-4939-9018-4_8

Romano-Armada, N., Yañez Yazlle, M. F., Irazusta, V., Rajal, V. B., and Moraga, N. B. (2020). Potential of bioremediation and PGP traits in streptomyces as strategies for bio-reclamation of salt-affected soils for agriculture. *Pathogens* 9:117. doi: 10.3390/pathogens9020117

Sáenz-Mata, J., Palacio-Rodríguez, R., Sánchez- Galván, H., and Balagurusamy, N. (2016). "Plant growth promoting rhizobacteria associated to halophytes: potential applications in agriculture," in *Sabkha Ecosystems*, eds M. Ajmal Khan, B. Boër, M. Öztürk, M. Clüsener-Godt, B. Gul, and S. W. Breckle (Cham: Springer International Publishing), 411–425. doi: 10.1007/978-3-319-27093-7_24

Schindelin, J., Arganda-Carreras, I., Frise, E., Kaynig, V., Longair, M., Pietzsch, T., et al. (2012). Fiji: an open-source platform for biological-image analysis. *Nat. Methods* 9, 676–682. doi: 10.1038/nmeth.2019

Schwyn, B., and Neilands, B. (1987). Universal chemical assay for the detection determination of siderophores. *Anal. Biochem.* 160, 47–56. doi: 10.1016/0003-2697(87)90612-9

Shankar, V., and Evelin, H. (2019). "Strategies for reclamation of saline soils," in *Microorganisms in Saline Environments: Strategies and Functions*, eds B. Giri and A. Varma (Cham: Springer Nature Switzerland AG), 439–449. doi: 10.1007/978-3-030-18975-4_19

Shekhar Nautiyal, C. (1999). An efficient microbiological growth medium for screening phosphate solubilizing microorganisms. *FEMS Microbiol. Lett.* 170, 265–270. doi: 10.1111/j.1574-6968.1999.tb13383.x

Srivastava, P., Wu, Q. S., and Giri, B. (2019). "Salinity : an overview," in *Microorganisms in Saline Environments: Strategies and Functions*, eds B. Giri and A. Varma (Cham: Springer Nature Switzerland AG 2019 B.), 3–18. doi: 10.1007/978-3-030-18975-4_1

Stefanello, R., Neves, L. A., Abbad, M. A., and Viana, B. B. (2015). Resposta fisiológica de sementes de chia (*Salvia hispanica* – Lamiales: Lamiaceae) ao estresse salino. *Biotemas* 28:35. doi: 10.5007/2175-7925.2015v28n4p35

Sugiura, D., and Tateno, M. (2011). Optimal leaf-to-root ratio and leaf nitrogen content determined by light and nitrogen availabilities. *PLoS ONE* 6:e22236. doi: 10.1371/journal.pone.0022236

Tabatabaei, S., Ehsanzadeh, P., Etesami, H., Alikhani, H. A., and Glick, B. R. (2016). Indole-3-acetic acid (IAA) producing pseudomonas isolates inhibit seed germination and α-amylase activity in durum wheat (*triticum turgidum* L.). *Spanish J. Agric. Res.* 14, 1–10. doi: 10.5424/sjar/2016141-8859

Thompson, L., and Troeh, F. (2002). *Suelos y Fertilidad*, *4th Edn*. Spain: Editorial Reverte SA.

Turan, M., Kitir, N., Elkoca, E., Uras, D., Ünek, C., Nikerel, E., et al. (2017). "Nonsymbiotic and symbiotic bacteria efficiency for legume growth under different stress conditions," in *Microbes for Legume Improvement*, eds A. Zaidi, M. S. Khan, and J. Musarrat (Cham: Springer International Publishing), 387–404. doi: 10.1007/978-3-319-59174-2_16

Vacheron, J., Desbrosses, G., Bouffaud, M. L., Touraine, B., Moënne-Loccoz, Y., Muller, D., et al. (2013). Plant growth-promoting rhizobacteria and root system functioning. *Front. Plant Sci.* 4:356. doi: 10.3389/fpls.2013.00356

Verma, P., Yadav, A. N., Khannam, K. S., Kumar, S., Saxena, A. K., and Suman, A. (2016). Molecular diversity and multifarious plant growth promoting attributes of Bacilli associated with wheat (*Triticum aestivum* L.) rhizosphere from six diverse agro-ecological zones of India. *J. Basic Microbiol.* 56, 44–58. doi: 10.1002/jobm.201500459

Wu, X., Xie, Y., Qiao, J., Chai, S., and Chen, L. (2019). Rhizobacteria strain from a hypersaline environment promotes plant growth of Kengyilia thoroldiana. *Microbiology* 88, 220–231. doi: 10.1134/S0026261719020127

Xiong, Y. W., Gong, Y., Li, X. W., Chen, P., Ju, X. Y., Zhang, C. M., et al. (2019). Enhancement of growth and salt tolerance of tomato seedlings by a natural halotolerant actinobacterium Glutamicibacter halophytocola KLBMP 5180 isolated from a coastal halophyte. *Plant Soil* 445, 307–322. doi: 10.1007/s11104-019-04310-8

Zhu, F., Qu, L., Hong, X., and Sun, X. (2011). Isolation and characterization of a phosphate- solubilizing halophilic Bacterium Kushneria sp. YCWA18 from daqiao saltern on the coast of yellow sea of China. *Evid. Based Complement. Altern. Med.* 2011:615032. doi: 10.1155/2011/615032

Conflict of Interest: The authors declare that the research was conducted in the absence of any commercial or financial relationships that could be construed as a potential conflict of interest.

Copyright © 2021 Yañez-Yazlle, Romano-Armada, Rajal and Irazusta. This is an open-access article distributed under the terms of the Creative Commons Attribution License (CC BY). The use, distribution or reproduction in other forums is permitted, provided the original author(s) and the copyright owner(s) are credited and that the original publication in this journal is cited, in accordance with accepted academic practice. No use, distribution or reproduction is permitted which does not comply with these terms.
