# Supplementary material for: Discerning Transcriptomic and Biochemical Responses of Arabidopsis thaliana Treated with the Biofertilizer Strain Priestia megaterium YC4-R4: Boosting Plant Central and Secondary Metabolism
Source: Plants (Basel). 2022 Nov 10;11(22):3039. doi: 10.3390/plants11223039 (PMC9697256; doi:10.3390/plants11223039)
Supplement: Supplementary file 1 [file plants-11-03039-s001.zip › Ref. 70.pdf]

DOI: 10.5586/asbp.3583

**Publication history**
Received: 2018-01-25
Accepted: 2018-05-12
Published: 2018-06-29

**Handling editor**
Grzegorz Jackowski, Faculty of Biology, Adam Mickiewicz University in Poznań, Poland

**Funding**
This article has received financial support from the Polish Ministry of Science and Higher Education under subsidy for maintaining the research potential of the Faculty of Biology and Chemistry, University of Bialystok.

**Competing interests**
No competing interests have been declared.

**Copyright notice**
© The Author(s) 2018. This is an Open Access article distributed under the terms of the Creative Commons Attribution License, which permits redistribution, commercial and noncommercial, provided that the article is properly cited.

**Citation**
Ciereszko I. Regulatory roles of sugars in plant growth and development. Acta Soc Bot Pol. 2018;87(2):3583. https://doi.org/10.5586/asbp.3583

**Digital signature**
This PDF has been certified using digital signature with a trusted timestamp to assure its origin and integrity. A verification trust dialog appears on the PDF document when it is opened in a compatible PDF reader. Certificate properties provide further details such as certification time and a signing reason in case any alterations made to the final content. If the certificate is missing or invalid it is recommended to verify the article on the journal website.

REVIEW

# Regulatory roles of sugars in plant growth and development

Iwona Ciereszko*

Institute of Biology, University of Bialystok, Ciolkowskiego 1J, 15-245 Bialystok, Poland

* Email: icier@uwb.edu.pl

## Abstract

In recent years, several studies have focused on the factors and mechanisms that regulate plant growth and development, as well as the functioning of signaling pathways in plant cells, unraveling the involvement of sugars in the processes regulating such growth and development. Saccharides play an important role in the life of plants: they are structural and storage substances, respiratory substrates, and intermediate metabolites of many biochemical processes. Sucrose is the major transport form of assimilates in plants. Sugars can also play an important role in the defense reactions of plants. However, it has been shown that glucose, sucrose, or trehalose-6-phosphate (Tre6P) can regulate a number of growth and metabolic processes, acting independently of the basal functions; they can also act as signaling molecules. Changes in the concentration, qualitative composition, and transport of sugars occur continuously in plant tissues, during the day and night, as well as during subsequent developmental stages. Plants have developed an efficient system of perception and transmission of signals induced by lower or higher sugar availability. Changes in their concentration affect cell division, germination, vegetative growth, flowering, and aging processes, often independently of the metabolic functions. Currently, the mechanisms of growth regulation in plants, dependent on the access to sugars, are being increasingly recognized. The plant growth stimulating system includes hexokinase (as a glucose sensor), trehalose-6-phosphate, and TOR protein kinase; the lack of Tre6P or TOR kinase inhibits the growth of plants and their transition to the generative phase. It is believed that the plant growth inhibition system consists of SnRK1 protein kinases and C/S1 bZIP transcription factors. The signal transduction routes induced by sugars interact with other pathways in plant tissues (for example, hormonal pathways) creating a complex communication and signaling network in plants that precisely controls plant growth and development.

## Keywords

glucose; receptor; SnRK; sucrose; sugar signaling; TOR; Tre6P

## Introduction

The growth and development of plants is regulated by various factors at different levels. Much research has been recently devoted to deciphering the mechanisms of growth, developmental regulation, and interactions between different signaling pathways in plant cells [1–8]. One of the most interesting aspects has been the demonstration of the regulatory function of sugars – molecules known for a long time to be involved in basal cell metabolism, and which are substrates or products of numerous chemical reactions.

The saccharides in plant tissues are primarily produced from triose-phosphates formed during photosynthesis occurring in leaves. Sucrose and starch, the final products of photosynthesis, can be temporarily stored in leaves, but most of the sucrose pool is transported to the acceptor tissues that do not produce this sugar [9–11]. The

2016;540:574–578. https://doi.org/10.1038/nature20591

94. Bledsoe SW, Henry C, Griffiths CA, Paul MJ, Feil R, Lunn JE, et al. The role of Tre6P and SnRK1 in maize early kernel development and events leading to stress-induced kernel abortion. BMC Plant Biol. 2017;17:74. http://doi.org/10.1186/s12870-017-1018-2
95. Jossier M, Bouly JP, Meimoun P, Arjmand A, Lessard P, Hawley S, et al. SnRK1 (SNF1-related kinase1) has a central role in sugar and ABA signalling in *Arabidopsis thaliana*. Plant J. 2009;59:316–328. https://doi.org/10.1111/j.1365-313X.2009.03871.x
96. Bai J, Mao J, Yang H, Khan A, Fan A, Liu S, et al. Sucrose non-ferment 1 related protein kinase 2 (*SnRK2*) genes could mediate the stress responses in potato (*Solanum tuberosum* L.). BMC Genetics. 2017;18:41. https://doi.org/10.1186/s12863-017-0506-6
97. Simon NM, Kusakina J, Fernández-López Á, Chembath A, Belbin FE, Dodd AN. The energy-signalling hub SnRK1 is important for sucrose-induced hypocotyl elongation. Plant Physiol. 2018;176:1299–1310. https://doi.org/10.1104/pp.17.01395
98. Xiong Y, Sheen J. Novel links in the plant TOR kinase signaling network. Curr Opin Plant Biol. 2015;28:83–91. https://doi.org/10.1016/j.pbi.2015.09.006
99. Dobrenel T, Caldana C, Hanson J, Robaglia C, Vincentz M, Veit B, et al. TOR signaling and nutrient sensing. Annu Rev Plant Biol. 2016;67:261–285. https://doi.org/10.1146/annurev-arplant-043014-114648
100. Li X, Cai W, Liu Y, Li H, Fu L, Liu Z, et al. Differential TOR activation and cell proliferation in *Arabidopsis* root and shoot apexes. Proc Natl Acad Sci USA. 2017;114:2765–2770. https://doi.org/10.1073/pnas.1618782114
101. Lee K, Seo PJ. *Arabidopsis* TOR signaling is essential for sugar-regulated callus formation. J Integr Plant Biol. 2017;59:742–746. https://doi.org/10.1111/jipb.12560
102. Schepetilnikov M, Ryabova LA. Recent discoveries on the role of TOR (target of rapamycin) signaling in translation in plants. Plant Physiol. 2018;176:1095–1105. https://doi.org/10.1104/pp.17.01243
103. Gupta A, Singh M, Laxmi A. Multiple interactions between glucose and brassinosteroid signal transduction pathways in *Arabidopsis* are uncovered by whole-genome transcriptional profiling. Plant Physiol. 2015;168:1091–1105. https://doi.org/10.1104/pp.15.00495
104. Zhang Y, He J. Sugar-induced plant growth is dependent on brassinosteroids. Plant Signal Behav. 2015;10(12):e1082700. https://doi.org/10.1080/15592324.2015.1082700
105. Djami-Tchatchou AT, Sanan-Mishra N, Ntushelo K, Dubery IA. Functional roles of microRNAs in agronomically important plants – potential as targets for crop improvement and protection. Front Plant Sci. 2017;8:378. https://doi.org/10.3389/fpls.2017.00378
106. Huang H, Long J, Zheng L, Li Y, Hu Y, Yu G, et al. Identification and characterization of microRNAs in maize endosperm response to exogenous sucrose using small RNA sequencing. Genomics. 2016;108:216–223. https://doi.org/10.1016/j.ygeno.2016.10.007
