# Supplementary material for: Discerning Transcriptomic and Biochemical Responses of Arabidopsis thaliana Treated with the Biofertilizer Strain Priestia megaterium YC4-R4: Boosting Plant Central and Secondary Metabolism
Source: Plants (Basel). 2022 Nov 10;11(22):3039. doi: 10.3390/plants11223039 (PMC9697256; doi:10.3390/plants11223039)
Supplement: Supplementary file 1 [file plants-11-03039-s001.zip › Ref. 75.pdf]

frontiers in Plant Science

ORIGINAL RESEARCH
published: 16 August 2021
doi: 10.3389/fpls.2021.710754

Check for updates

Fine-Tuning Florigen Increases Field Yield Through Improving Photosynthesis in Soybean

Kun Xu[1,2†], Xiao-Mei Zhang[1†], Haifeng Chen[3†], Chanjuan Zhang[3], Jinlong Zhu[1,2], Zhiyuan Cheng[1], Penghui Huang[1], Xinan Zhou[3], Yuchen Miao[4], Xianzhong Feng[5]* and Yong-Fu Fu[1]*

[1] MOA Key Laboratory of Soybean Biology, National Key Facility of Crop Gene Resource and Genetic Improvement, Institute of Crop Sciences, Chinese Academy of Agricultural Sciences, Beijing, China, [2] Key Laboratory of Soybean Molecular Design Breeding, Northeast Institute of Geography and Agroecology, The Innovative Academy of Seed Design, Chinese Academy of Sciences, Harbin, China, [3] Oil Crops Research Institute, Chinese Academy of Agricultural Sciences, Key Laboratory of Biology and Genetic Improvement of Oil Crops, Ministry of Agriculture, Wuhan, China, [4] Key Laboratory of Plant Stress Biology, State Key Laboratory of Cotton Biology, School of Life Sciences, Henan University, Kaifeng, China, [5] CAS Key Laboratory of Soybean Molecular Design Breeding, Northeast Institute of Geography and Agroecology, Chinese Academy of Sciences, Changchun, China

OPEN ACCESS

Edited by:
Scott A. Boden,
John Innes Centre, United Kingdom

Reviewed by:
Kyuya Harada,
Osaka University, Japan
Yaroslav B. Blume,
National Academy of Sciences of Ukraine (NAN Ukraine), Ukraine

***Correspondence:**
Xianzhong Feng
fengxianzhong@iga.ac.cn
Yong-Fu Fu
fuyongfu@caas.cn

† These authors have contributed equally to this work

Specialty section:
This article was submitted to Technical Advances in Plant Science, a section of the journal Frontiers in Plant Science

Received: 17 May 2021
Accepted: 28 June 2021
Published: 16 August 2021

Citation:
Xu K, Zhang X-M, Chen H, Zhang C, Zhu J, Cheng Z, Huang P, Zhou X, Miao Y, Feng X and Fu Y-F (2021) Fine-Tuning Florigen Increases Field Yield Through Improving Photosynthesis in Soybean. Front. Plant Sci. 12:710754. doi: 10.3389/fpls.2021.710754

Crop yield has been maintaining its attraction for researchers because of the demand of global population growth. Mutation of flowering activators, such as florigen, increases plant biomass at the expense of later flowering, which prevents crop maturity in the field. As a result, it is difficult to apply flowering activators in agriculture production. Here, we developed a strategy to utilize florigen to significantly improve soybean yield in the field. Through the screening of transgenic lines of RNAi-silenced florigen homologs in soybean (*Glycine-max-Flowering Locus T Like*, *GmFTL*), we identified a line, *GmFTL*-RNAi#1, with minor changes in both *GmFTL* expression and flowering time but with notable increase in soybean yield. As expected, *GmFTL*-RNAi#1 matured normally in the field and exhibited markedly high yield over multiple locations and years, indicating that it is possible to reach a trade-off between flowering time and high yield through the fine-tuning expression of flowering activators. Further studies uncovered an unknown mechanism by which *GmFTL* negatively regulates photosynthesis, a substantial source of crop yield, demonstrating a novel function of florigen. Thus, because of the highly conserved functions of florigen in plants and the classical RNAi approach, the findings provide a promising strategy to harness early flowering genes to improve crop yield.

Keywords: high yield, florigen, *FT*, photosynthesis, soybean, vegetative growth

INTRODUCTION

The global crop demand for human consumption and livestock feed is forecasted to increase by 110% from 2005 to 2050 (Tilman et al., 2011). However, advances in breeding, genomics, and transgenic technology are predicted to increase yield by up to 20% (Long et al., 2015). Therefore, there is an urgent need to develop innovative approaches to increase crop yields. Photosynthesis provides a substantial means to adjust crop yields (Ort et al., 2015). Regulation of photosynthesis happens at multiple levels (Long et al., 2006; Parry et al., 2007), which could serve as targets to

hypocotyl in *Arabidopsis thaliana*. *Plant Mol. Biol.* 71, 39–50. doi: 10.1007/s11103-009-9507-9

Yoo, S. K., Chung, K. S., Kim, J., Lee, J. H., Hong, S. M., Yoo, S. J., et al. (2005). CONSTANS activates SUPPRESSOR OF OVEREXPRESSION OF CONSTANS 1 through FLOWERING LOCUS T to promote flowering in Arabidopsis. *Plant Physiol.* 139, 770–778. doi: 10.1104/pp.105.066928

Zhang, L., Wang, R., and Hesketh, J. D. (2001). Effects of photoperiod on growth and development of soybean floral bud in different maturity. *Agron. J.* 93, 944–948. doi: 10.2134/agronj2001.934944x

Conflict of Interest: The authors declare that the research was conducted in the absence of any commercial or financial relationships that could be construed as a potential conflict of interest.

Publisher's Note: All claims expressed in this article are solely those of the authors and do not necessarily represent those of their affiliated organizations, or those of the publisher, the editors and the reviewers. Any product that may be evaluated in this article, or claim that may be made by its manufacturer, is not guaranteed or endorsed by the publisher.

Copyright © 2021 Xu, Zhang, Chen, Zhang, Zhu, Cheng, Huang, Zhou, Miao, Feng and Fu. This is an open-access article distributed under the terms of the Creative Commons Attribution License (CC BY). The use, distribution or reproduction in other forums is permitted, provided the original author(s) and the copyright owner(s) are credited and that the original publication in this journal is cited, in accordance with accepted academic practice. No use, distribution or reproduction is permitted which does not comply with these terms.
