# Supplementary material for: Discerning Transcriptomic and Biochemical Responses of Arabidopsis thaliana Treated with the Biofertilizer Strain Priestia megaterium YC4-R4: Boosting Plant Central and Secondary Metabolism
Source: Plants (Basel). 2022 Nov 10;11(22):3039. doi: 10.3390/plants11223039 (PMC9697256; doi:10.3390/plants11223039)
Supplement: Supplementary file 1 [file plants-11-03039-s001.zip › Table S1. DEGs considered in this study.pdf]

Table S1. Upregulated genes in RNA-seq analysis. Genes with a minimal of 0.7 in log2FC

Gene ID	log2FC	p-value	FDR	Gene Type
**AT2G24980**	1,8400	1,15E-12	1,13E-10	Proline-rich extensin-like
**AT3G60140**	1,6709	9,45E-32	8,83E-29	Glycosyl hydrolase
**AT4G25820**	1,5949	1,06E-16	1,85E-14	Xyloglucan endotransglucosylase/hydrolase 14
**AT3G24460**	1,4744	1,13E-28	8,21E-26	Sphingolipid biosynthesis protein
**AT5G24150**	1,4604	4,56E-61	4,47E-57	FAD/NAD(P)-binding oxidoreductase
**AT3G44300**	1,4254	2,06E-46	4,04E-43	Nitrilase 2
**AT5G06640**	1,3736	5,26E-09	2,80E-07	Proline-rich extensin-like
**AT5G08640**	1,3403	1,22E-23	4,90E-21	Flavonol synthase 1
**AT5G56080**	1,2733	1,34E-09	8,07E-08	Nicotianamine synthase 2
**AT1G64780**	1,2690	2,91E-21	8,92E-19	Ammonium transporter 1;2
**AT3G21670**	1,2036	3,02E-38	3,48E-35	Major facilitator
**AT1G32900**	1,1899	7,58E-40	9,30E-37	UDP-Glycosyltransferase
**AT1G65060**	1,1446	3,76E-25	1,94E-22	4-coumarate:CoA ligase 3
**AT4G19690**	1,1446	4,63E-15	6,63E-13	Iron-regulated transporter 1
**AT5G45070**	1,0757	7,87E-11	5,55E-09	Phloem protein 2-A8
**AT5G04950**	1,0591	1,43E-24	6,86E-22	Nicotianamine synthase 1
**AT2G32530**	1,0507	9,77E-17	1,73E-14	Cellulose synthase-like B3
**AT4G16590**	1,0260	3,84E-11	2,84E-09	Cellulose synthase-like A01
**AT4G10120**	1,0234	8,89E-30	7,27E-27	Sucrose-phosphate synthase
**AT1G01580**	1,0217	5,31E-15	7,55E-13	Ferric reduction oxidase 2
**AT3G54590**	0,9654	3,53E-08	1,59E-06	Hydroxyproline-rich glycoprotein
**AT3G44990**	0,9639	1,39E-25	7,34E-23	Xyloglucan endo-transglycosylase-related 8
**AT4G12320**	0,9598	9,20E-28	5,82E-25	Cytochrome P450
**AT2G32270**	0,9347	4,76E-05	9,69E-04	Zinc transporter 3 precursor
**AT2G41290**	0,9298	2,22E-07	8,44E-06	Strictosidine synthase-like 2
**AT4G27030**	0,9260	1,38E-13	1,57E-11	Fatty acid desaturase A
**AT1G60590**	0,9208	1,16E-10	7,95E-09	Pectin lyase-like
**AT4G24000**	0,9068	4,10E-11	3,02E-09	Cellulose synthase like G2
**AT1G62180**	0,8843	3,44E-23	1,30E-20	5'adenylylphosphosulfate reductase 2
**AT5G58770**	0,8634	1,87E-16	3,14E-14	Undecaprenyl pyrophosphate synthetase
**AT1G61120**	0,8215	4,96E-19	1,26E-16	Terpene synthase 04
**AT5G07010**	0,8187	7,93E-13	8,02E-11	Sulfotransferase 2A
**AT4G26850**	0,8161	8,75E-22	2,86E-19	Mannose-1-phosphate guanylyltransferase
**AT3G21560**	0,8007	2,09E-20	6,12E-18	UDP-Glycosyltransferase
**AT3G24300**	0,7905	3,23E-07	1,18E-05	Ammonium transporter 1;3
**AT5G43630**	0,7870	4,51E-17	8,50E-15	Zinc knuckle (CCHC-type)
**AT4G14090**	0,7838	2,31E-10	1,53E-08	UDP-Glycosyltransferase
**AT5G43350**	0,7813	5,56E-09	2,95E-07	Phosphate transporter 1;1
**AT4G15550**	0,7781	6,90E-17	1,24E-14	Indole-3-acetate beta-D-glucosyltransferase
**AT1G47510**	0,7769	4,21E-04	6,04E-03	Inositol polyphosphate 5-phosphatase 11
**AT3G46670**	0,7729	1,12E-11	9,07E-10	UDP-glucosyl transferase 76E11
**AT2G23010**	0,7649	1,02E-09	6,24E-08	Aerine carboxypeptidase-like 9
**AT2G23130**	0,7268	6,50E-09	3,42E-07	Arabinogalactan protein 17
**AT3G52720**	0,7257	4,13E-15	5,96E-13	Alpha carbonic anhydrase 1
**AT5G55380**	0,7243	3,43E-05	7,26E-04	Membrane bound O-acyl transferase
**AT1G56430**	0,7229	1,05E-12	1,04E-10	Nicotianamine synthase 4
**AT2G32540**	0,7196	2,35E-14	3,03E-12	Cellulose synthase-like B4
**AT2G25680**	0,7154	1,91E-06	5,73E-05	Molybdate transporter 1
**AT5G17300**	0,7134	1,12E-12	1,11E-10	Homeodomain-like
**AT3G45130**	0,7065	1,67E-05	3,86E-04	Lanosterol synthase 1
