# Supplementary figures and images for: Discerning Transcriptomic and Biochemical Responses of Arabidopsis thaliana Treated with the Biofertilizer Strain Priestia megaterium YC4-R4: Boosting Plant Central and Secondary Metabolism
Source: Plants (Basel). 2022 Nov 10;11(22):3039. doi: 10.3390/plants11223039 (PMC9697256; doi:10.3390/plants11223039)

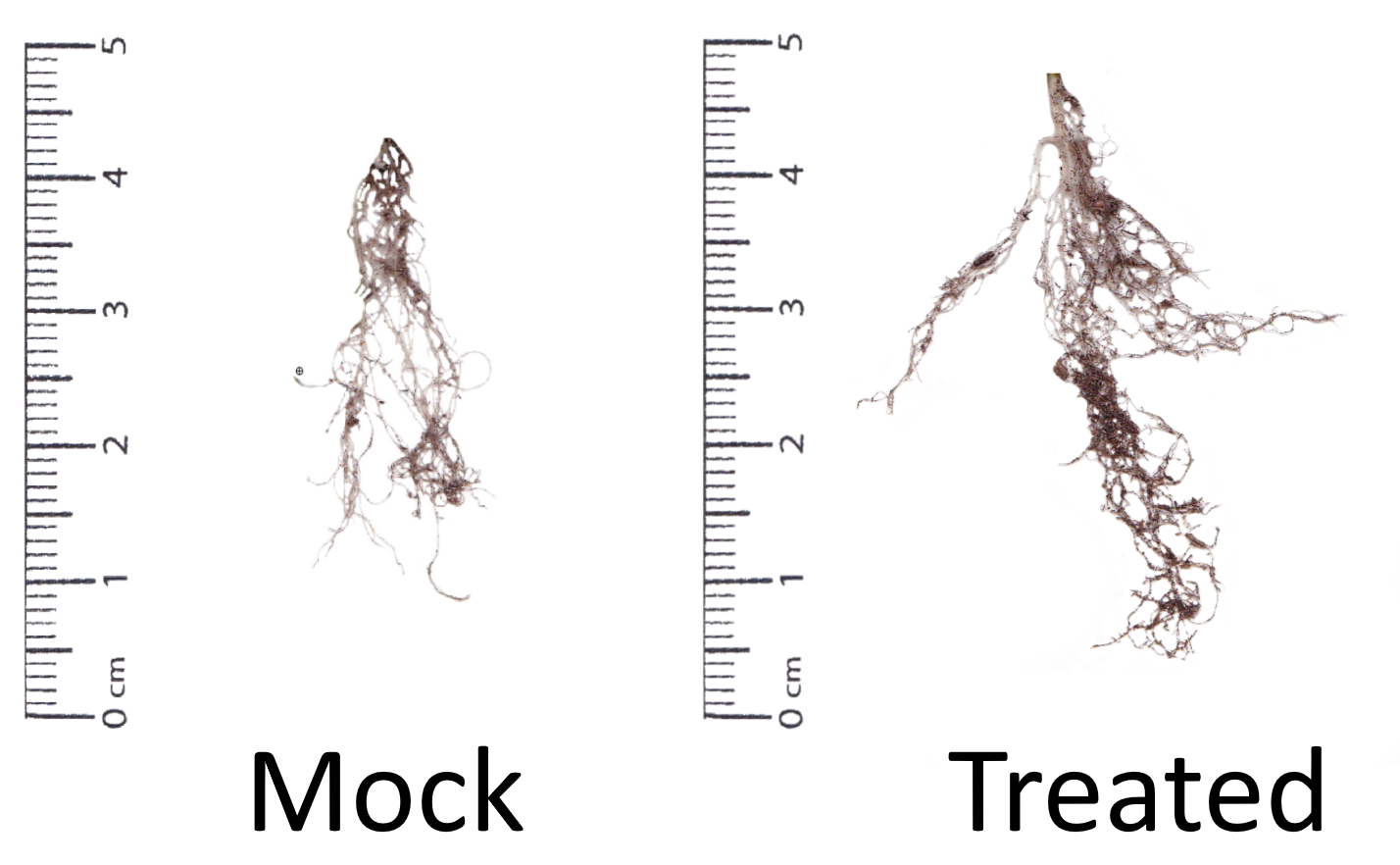

Supplement: Supplementary file 1 [file plants-11-03039-s001.zip › Supplementary Figure S1. Root phenotyping after treatment.tiff]

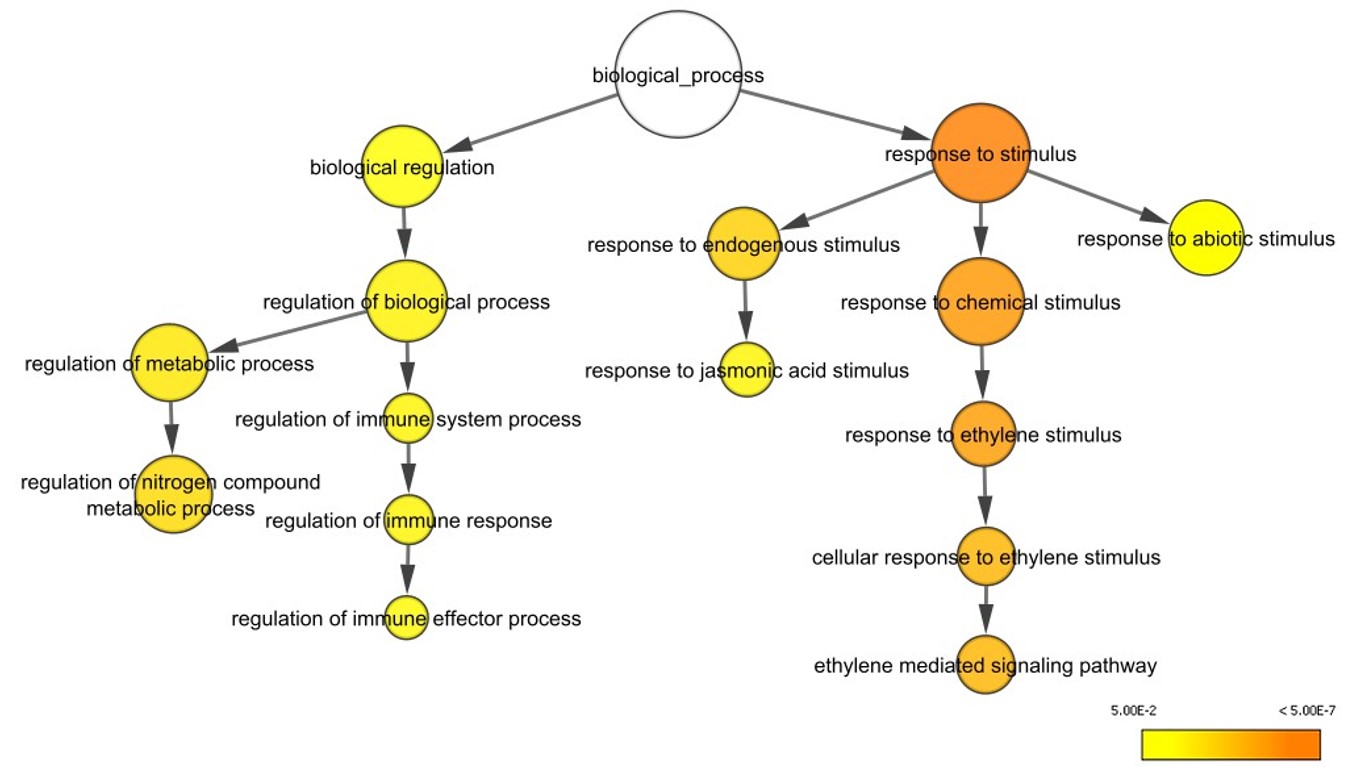

Supplement: Supplementary file 1 [file plants-11-03039-s001.zip › Supplementary Figure S2. RNA-seq downregulated DEGs.jpg]

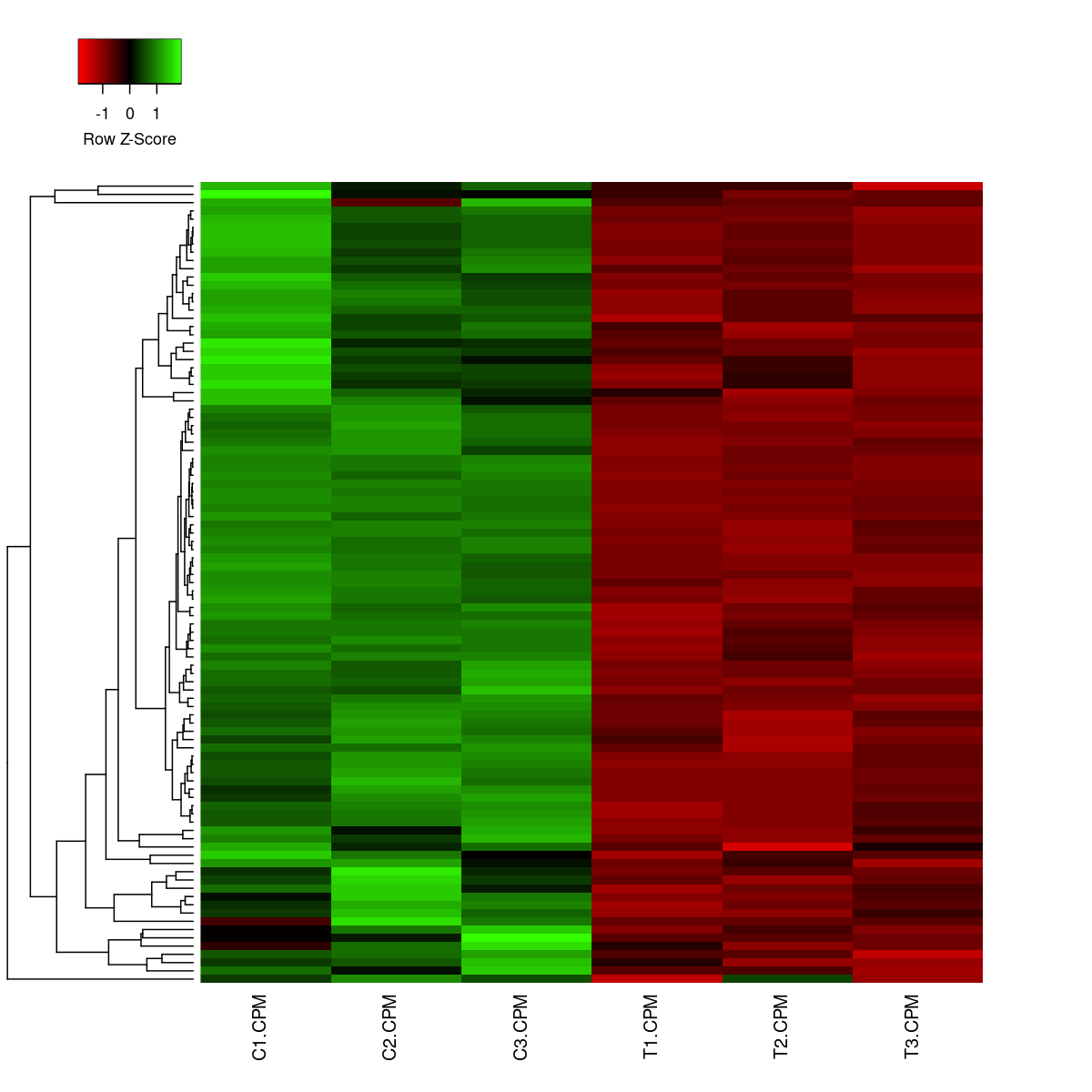

Supplement: Supplementary file 1 [file plants-11-03039-s001.zip › Supplementary Figure S3. Downregulated DGEs Heatmap.tiff]

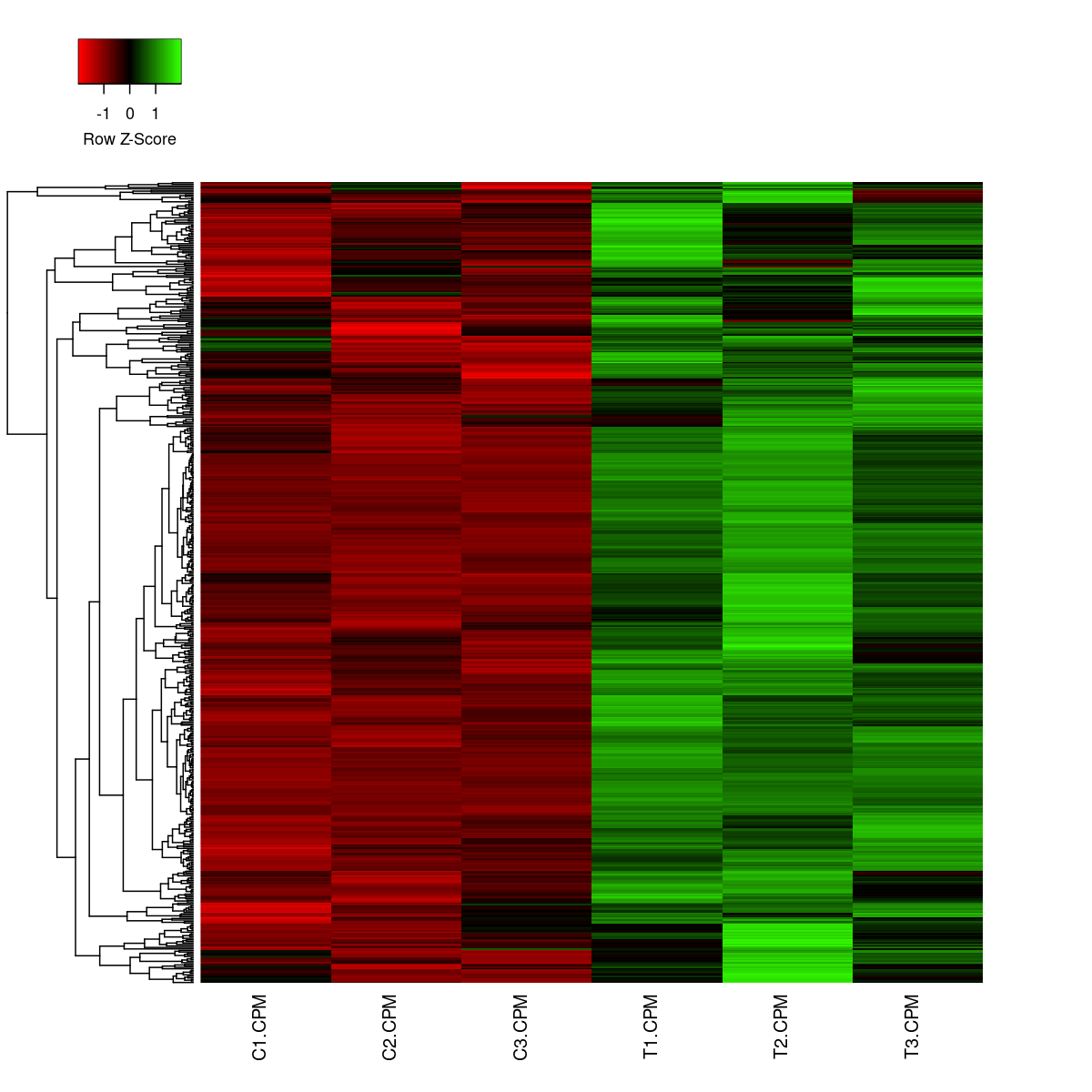

Supplement: Supplementary file 1 [file plants-11-03039-s001.zip › Supplementary Figure S4. Upregulated DEGs Heatmap.tiff]
